# Burden of Depression among Working-Age Adults with Rheumatoid Arthritis

**DOI:** 10.1155/2018/8463632

**Published:** 2018-06-03

**Authors:** Arijita Deb, Nilanjana Dwibedi, Traci LeMasters, Jo Ann Hornsby, Wenhui Wei, Usha Sambamoorthi

**Affiliations:** ^1^School of Pharmacy, Department of Pharmaceutical Systems and Policy, West Virginia University, Morgantown, WV, USA; ^2^School of Medicine, West Virginia University, Morgantown, WV, USA; ^3^Regeneron Pharmaceuticals, Tarrytown, NJ, USA

## Abstract

**Objective:**

This study estimated the excess clinical, humanistic, and economic burden associated with depression among working-age adults with Rheumatoid Arthritis (RA).

**Methods:**

A retrospective cross-sectional study was conducted among working-age (18 to 64 years) RA patients with depression (*N* = 647) and without depression (*N* = 2,015) using data from the nationally representative Medical Expenditure Panel Survey for the years 2009, 2011, 2013, and 2015.

**Results:**

Overall, 25.8% had depression. In adjusted analyses, adults with RA and depression compared to those without depression were significantly more likely to have pain interference with normal work (severe pain: AOR = 2.22; 95% CI = 1.55, 3.18), functional limitations (AOR = 2.17; 95% CI = 1.61, 2.94), and lower mental health HRQoL scores. Adults with RA and depression had significantly higher annual healthcare expenditures ($14,752 versus 10,541, *p* < .001) and out-of-pocket spending burden. Adults with RA and depression were more likely to be unemployed and among employed adults, those with depression had a significantly higher number of missed work days annually and higher lost annual wages due to missed work days.

**Conclusions:**

This study highlights the importance of effectively managing depression in routine clinical practice of RA patients to reduce pain and functional limitations, improve quality of life, and lower direct and indirect healthcare costs.

## 1. Introduction

Rheumatoid Arthritis (RA) is one of the most debilitating chronic conditions, with the onset often occurring during the prime working years of lives, between the ages of 20 and 40 years [1]. Individuals with RA experience substantial pain and RA causes permanent work disability in more than one-third of affected patients within 10 years of onset [2]. Such pain and disabilities associated with RA may contribute to a higher prevalence of depression in individuals with RA compared to healthy controls [3]. An expert review of depression in arthritis reported that the prevalence of depression in adults with RA can be as high as 66.2% [4]. A systematic review and meta-analysis of 72 studies estimated the prevalence rate to be 16.8% [5].

The disease burden of depression in RA can be substantial because depression can worsen survival [6] and increase morbidity in terms of disability, health-related quality of life (HRQoL), RA disease activity [7], and pain [8]. Depression in RA can also increase healthcare resource utilization [9, 10], which can lead to high health care expenditures for both insurance payers, patients and families. As depression in RA can aggravate disability, an individual may also suffer economic losses due to work inability or even experience economic losses due to missed work days. Although not specific to RA, one study estimated that 6.9 million working-age adults reported arthritis-attributable work limitation [11]. One can speculate that depression can worsen the work limitation because the presence of depression along with any chronic physical condition more than doubles the likelihood of work absenteeism as compared to the presence of any chronic physical condition without depression [12].

However, to date, no published study in the US has done a comprehensive analysis of the humanistic and economic burden associated with depression among RA patients, particularly among working-age adults. In fact, a recent review highlighted the significant knowledge gap in estimating the disease burden of depression in adults with arthritis [4]. Although there has been a handful of studies on the association between depression and HRQoL among adults with RA, most of these studies have been conducted outside the US [13, 14] or only among women in a specific setting [15]. One US study used a cross-sectional design and examined the relationship between depression and disability, HRQoL in the US with data from the 2011 Behavioral Risk Factor Surveillance System [16]. However, this study included all forms of arthritis and did not focus on RA. Again, only one study using 2006 commercial claims data in the US found that RA patients with depression had a significantly higher adjusted annual healthcare costs as compared to RA patients without depression ($12,225 versus $11,404) [9]. However, this study was based on commercially insured RA patients and may not be representative of the US national population. Furthermore, this commercial insurance data did not include certain patient characteristics which are important confounders of healthcare costs such as race, education, and income level.

Therefore, the objective of this study is to examine the incremental burden of depression on the clinical, humanistic, and economic outcomes among working-age adults with RA.

## 2. Methods

### 2.1. Study Design

A retrospective cross-sectional study design with data from a nationally representative sample of working-age adults (18–64 years) was used.

### 2.2. Data Source

We used data from the Medical Expenditure Panel Survey (MEPS), an annual household survey of the noninstitutionalized civilian population in the US. Information on demographic characteristics, medical conditions, health status, utilization of health care services, charges and payments, access to care, health insurance coverage, income, education, employment, and missed workdays of the participants in the survey were extracted from the household component of MEPS. We pooled four years of data (2009, 2011, 2013, and 2015) to have sufficient sample size and used data from alternate years to avoid including two observations per individual. Furthermore, a question related to the type of arthritis was available in these years. MEPS recommends pooling of data to increase sample size and it is a common practice in published literature with MEPS data [17].

### 2.3. Study Sample

**Figure 1:**
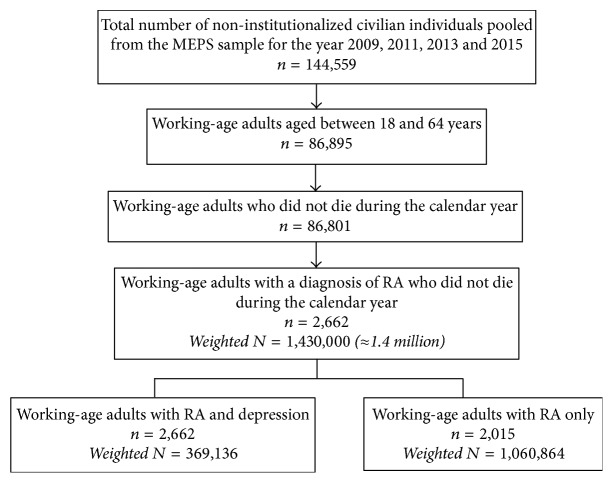
Flow diagram of study sample.

The study sample (*N* = 2,662) consisted of working-age (18–64 years) who were diagnosed with RA and who were alive during the study period (2009, 2011, 2013, and 2015) (Figure 1). RA was identified from the priority condition enumeration section. In this section, respondents were queried whether an individual in the household has ever been told by a doctor or another health professional that she/he had arthritis and type of arthritis (RA versus osteoarthritis). We also identified RA from medical condition file with the clinical classification code (202). Medical conditions were reported by the respondents if they sought treatment for the condition, or if the condition resulted in disability, or if the condition was bothersome. The responses were recorded as texts, and these texts were translated into International Classification of Diseases, 9th Edition, Clinical Modification (ICD-9-CM) codes by professional coders. In addition, MEPS data provides clinical classification codes, which are aggregated ICD-9-CM codes into clinically meaningful categories that group similar conditions (Agency for Healthcare Research and Quality).

### 2.4. Conceptual Framework

The conceptual framework for this study was adapted from the Andersen's Expanded Behavioral Model which posits that health services utilization and outcomes of an individual are a function of predisposing factors (e.g., age, sex, and race), enabling factors (e.g., marital status, education and poverty status), need factors (e.g., chronic conditions, health status), and personal health practices (e.g., physical activity, obesity, and smoking) [18].

### 2.5. Measures

#### 2.5.1. Clinical Outcomes

*Pain Interference with Normal Activities*. Based on a self-administered single-item question, pain interference with normal activities during the past four weeks among the household respondents was measured. The answers were recorded on a 5-point Likert scale during the past 4 weeks. In MEPS, pain was reported on a 5-point scale: (1) not at all, (2) a little bit, (3) moderately, (4) quite a bit, and (5) extremely. For purposes of this study we group pain categories as follows: (1) not at all/little bit; (2) moderate; (3) severe (quite a bit/extremely). Self-reported pain from MEPS has been used in published literature to estimate the cost of pain [19]. There were 42 individuals (5.9%) in the depression group and 140 individuals (6.6%) in the no depression group with missing data on pain inference variable. These individuals were not included in the analysis.

*Any Functional Limitations*. This variable summarizes whether an individual had any limitations in instrumental activities of daily living (IADL) (e.g., shopping, cooking, using phone, paying bills, taking medications, driving, doing laundry, or going shopping), activities of daily living (ADL) (e.g., bathing, dressing, grooming, mouthcare, toileting, and eating), functional limitations (walking, climbing stairs, grasping objects, reaching overhead, lifting, bending or stooping, or standing for long periods of time), or activity limitations (work, housework, or school).

#### 2.5.2. Humanistic Outcome: Health-Related Quality of Life

HRQoL was measured by the generic Short-Form-12 Version 2 (SF12-V2) summary scores. The SF12-V2 is a generic multipurpose survey with 12 questions, which encompass eight domains (role physical, role emotional, physical function, social function, mental health, vitality, pain, and general health). These questions are designed to provide summary measures of overall HRQoL of an individual. The* Mental Component Summary (MCS) score *was derived from the responses to the items in the domains: vitality, social functioning, role emotional (limitations in work and daily activities because of emotional problems), and mental health. The* Physical Component Summary* (PCS) score was derived from the responses to the items in the domains: physical functioning, role physical, bodily pain, and general health. Both MCS and PCS scores ranged from 0 to 100, with higher scores representing better self-reported health and better HRQoL related to mental or physical health [20].

#### 2.5.3. Economic Outcomes: Direct Healthcare Expenditures

*Total Healthcare Expenditures*. In the MEPS, expenditures are defined as the sum of direct payments for care provided during the year. The direct payments include twelve sources of payment categories such as out-of-pocket by patient or families, Medicare, Medicaid, Private Insurance, Veteran Administration, worker's compensation, and others. Total annual per person healthcare expenditures were calculated as the sum of inpatient, outpatient, emergency, dental, home health, vision, prescription drugs, and other medical supplies. All expenditures were inflation adjusted to 2015 US dollars (USD) using consumer price index for medical services from the bureau of medical services.

*Total Out-of-Pocket Spending Burden by Patients and Families.* We also estimated the total out-of-pocket spending on healthcare by the respondent and/or family. These included annual deductibles, copayment, and coinsurance for services and payment for services that were not covered by health insurance. We calculated out-of-pocket spending burden as the ratio of out-of-pocket healthcare expenditures to personal income [21], which varied from zero to 100. Based on published literature, we defined spending 10% or more of personal income on health care as high out-of-pocket spending burden [22].

#### 2.5.4. Economic Outcomes: Indirect Healthcare Burden

*Unemployment (i.e., Labor Market Outcome). *In the MEPS, employment section covers questions about each person's employment or self-employment status. Based on these questions, we classified individuals who were currently unemployed.

*Missed work days* were measured whether individuals lost a half-day or more from work because of illness, injury, or mental or emotional problems during the year and how many workdays were lost. This was calculated only for employed adults.

*Lost wages* for each individual were calculated by multiplying missed work days with an average daily wage of each individual. All wages were adjusted to 2015 general consumer inflation rates derived from the bureau of labor statistics.

*Key Explanatory Variable*

*Depression (Yes/No).* Depression was identified based on the clinical classification code “657,” which included both depressive disorders and bipolar disorders.

*Other Explanatory Variables. *Predisposing characteristics were sex (male, female), race/ethnicity (Whites, African-American, and other racial minorities), and age in years (18–39, 40–49, and 50–64). Enabling factors comprised marital status (married, widow, separated/divorced, and never married), family poverty status (not poor, poor), health insurance status (public, private), and usual source of care (yes, no). Need factors included having a chronic condition other than RA from a list of eight conditions (asthma, cancer, chronic obstructive pulmonary disease, diabetes, heart disease, hypertension, stroke, and thyroid), anxiety, perceived physical health status (excellent/very good, good, and fair/poor), and perceived mental health status (excellent/very good, good and fair/poor). Personal health practice factors included obesity (obese and not obese), smoking status (current smoker, others, and missing), and exercise (“yes” and “no” exercise).

### 2.6. Statistical Analyses

A variety of statistical analyses were used based on the measurement of the outcome variables. The unadjusted relationships between the presence of depression and categorical variables and outcomes (pain interference with activities, employment, and OOP burden) were assessed with chi-square tests. Unadjusted differences in continuous outcomes (PCS, MCS, all-cause healthcare expenditures, and out-of-pocket spending by the patients and their families) by depression were tested with *t*-tests. Multinomial logistic regression was used to analyze the association between depression and pain-related interference with normal work after adjusting for the predisposing, enabling, need, and external environment characteristics. Logistic regression was used to analyze the association between depression and binary categorical variables (e.g., any limitations, unemployment, and out-of-pocket spending burden) after adjusting for covariates. Adjusted models for continuous outcomes (expenditures, out-of-pocket expenditures, and lost wages) consisted of Generalized Linear Models (GLM). GLM is flexible and can handle categorical outcomes, continuous outcomes, and count-data with the appropriate distribution family and a link function. For count-data (e.g., the number of missed work days) we used negative binomial regression.

*Counterfactual Prediction Technique (Recycled Prediction).* We used counterfactual recycled prediction, an approach that is gaining attention [23, 24] to estimate excess total healthcare expenditures, prescription expenditures, missed work days, and lost wages attributable to depression among working-age adults with Rheumatoid Arthritis. The recycled prediction technique is a preferred approach because it adjusts for differences in characteristics between the depression and no depression group by creating counterfactual scenarios. In all recycled prediction models, confidence intervals were obtained using 2000 bootstrap replications using the percentile method. To account for the complex design of MEPS, we conducted all analyses using the survey procedures in Statistical Analysis Software (SAS) version 9.3, Cary, NC, USA, and the survey design features with STATA 14. As we pooled four years, to get annualized weighted numbers, we divided the weights by four, recommended by the MEPS investigators [25] and used in the published literature [26].

## 3. Results

### 3.1. Description of the Study Sample

Majority of the study sample was female (64%) and white (63%), aged between 50 and 64 years (61%), and had multimorbidity (72%). Only 26% of individuals who perceived themselves having excellent or very good physical health and 44.8% reported having excellent or very good mental health (see Table 1).

Overall, 25.8% of adults with RA reported depression (Table 2). We observed significant differences in the rate of depression by predisposing, enabling, need factors, and personal health practices except for age, education, and region. For example, female adults with RA reported a significantly higher rate of depression than their male counterparts (29.6% versus 19.1%). The higher rate of depression was also observed among individuals with multimorbidity (29.1% versus 17.4%). A higher percentage of those who perceived themselves to be poor/fair physical health reported depression compared to those in excellent or very good health (38.0% versus 12.4%).

### 3.2. Clinical Outcomes

#### 3.2.1. Pain Interference with Normal Activities

A higher percentage of adults with RA and depression reported severe pain interfering with work or other normal activities compared to those with RA and no depression (54.8% versus 30.8%) (Table 3). After adjusting for predisposing, enabling, need, personal health practices, and external environment factors, and adults with depression were 2.2 times as likely to report severe pain interference with normal work activities than those without depression (AOR = 2.22; 95% CI = 1.55, 3.18) (Table 3).

#### 3.2.2. Any Functional Limitations

A significantly higher percentage of adults with RA and depression reported any functional limitations compared to those with RA and no depression (79% versus 51.1%) (Table 3). After adjusting for covariates adults with RA and depression were more than 2 times as likely to report any functional limitations (AOR = 2.24; 95% CI = 1.62, 3.10) (Table 3).

### 3.3. Humanistic Outcomes

Adults with RA and depression reported significantly lower HRQoL scores in both Physical Component Summary score (35.1 versus 40.2, *p* < .001) and Mental Component Summary score (37.2 versus 48.7, *p* < .001) compared to adults with RA without depression (Table 4). In adjusted analyses, a significant difference was observed only in the mental domain of the HRQoL; the presence of depression was associated with a decrement of 8.72 in MCS scores (Table 4). The counterfactual predictions yielded similar differences in MCS (37.19 in adults with depression versus 45.91 in adults without depression, *p* < .001). The relationship between depression and PCS scores became insignificant after adjustment for the presence of multiple chronic conditions.

### 3.4. Economic Outcomes

#### 3.4.1. Direct Total Healthcare Expenditures

In unadjusted analysis, adults with RA and depression had significantly higher annual healthcare expenditures ($17,941 versus $10,064 *p* < .001). In the adjusted GLM with gamma distribution and log-link, we found that depression was associated with greater total healthcare expenditures compared to those without depression (Beta = 0.34, SE = 0.08). When converted to original dollars this represented $14,752 for those with depression and $10,541 for those without depression (Table 5). Estimates from counter-factual recycled prediction revealed that depression was associated with an excess of $4,212 total healthcare expenditures with 95% CI = $4,114, $4,318.

In unadjusted analysis, patients/families in the RA + depression group spent significantly higher amounts out-of-pocket on health care compared to the RA + no depression group ($1,443 versus $1,052, *p* < .001). In the adjusted GLM with gamma distribution and log-link, we found that depression was associated with greater total out-of-pocket healthcare spending compared to those without depression (Beta = 0.23, SE = 0.06). When converted to original dollars this represented $1,232 for those with depression and $979 for those without depression (Table 5). Estimates from counter-factual recycled prediction revealed that depression was associated with an excess of $253 with 95% CI = $247, 260.

When high out-of-pocket spending burden was measured as spending greater than 10% of income on healthcare, we found that 30.7% of adults with depression and 21.3% of adults without depression had high out-of-pocket spending burden. After adjusting for other factors, adults with depression were significantly more likely to have high out-of-pocket spending burden (AOR = 1.34; 95% CI = 1.01, 1.79).

#### 3.4.2. Indirect Economic Burden

*Labor Market Outcome (Unemployment), Missed Work Days, and Lost Wages. *Presence of depression was significantly associated with unemployment among adults with RA; 64.1% of adults with depression were unemployed compared to 40.1% adults without depression. Even after controlling for other factors mentioned in the methods section, adults with RA and depression were 1.55 times as likely as those without depression to be unemployed (AOR = 1.55; 95% CI = 1.14, 2.10). Among employed adults, those with depression had significantly higher number of missed work days annually (9 versus 6, *p* < .05) and higher lost wages ($813 versus $571, *p* < .05) due to missed work (Table 5). We obtained similar results with counterfactual recycled predictions.

## 4. Discussion

In this study using a nationally representative sample of community-dwelling US adults, one in four working-age adults with RA reported depression. This rate is considerably higher compared to the 6.8% rate of depression in the general population in the US [27] and higher than the pooled depression rate of 16.8% reported by Matcham and colleagues in a meta-analysis of 72 studies that included 13,189 RA patients [5]. The same meta-analysis also reported the presence of depressive symptoms in 38.8% of RA patients measured using Patient Health Questionnaire (PHQ-9) and 34.2% of RA patients measured using Hospital Anxiety and Depression Scale (HADS) [5]. Therefore, the differences in the rate of depression in RA patients can be explained by the differences in the instruments used to identify depression.

Our study findings indicated the substantial additional clinical burden imposed by depression in working-age adults with RA. These findings have implications for comanagement of depression and RA. Although not specific to RA, a randomized clinical trial of 1,001 patients with concurrent depression and arthritis and seeking care from 18 primary care clinics [28] suggested that collaborative depression care not only reduced depressive symptoms but also improved arthritis related outcomes, such as decreasing pain and functional limitations. There is some evidence that disease-modifying drugs used to treat RA can have spill-over effects in reducing depressive symptoms. For example, depression levels decreased significantly following commencement and continuity of rituximab, a B cell-directed therapy, among individuals with RA [29]. Therefore, future studies need to systematically evaluate whether antirheumatic treatment among individuals with RA can help alleviate depressive symptoms.

We also observed significant decrements in HRQoL measures, specifically the MCS scores. This is not surprising; however, it is important given the strong association between patient-reported outcomes and disease activity [30]. It has also been suggested that patient-reported outcomes such as the HRQoL and other measures in clinical trials and routine clinical practice may shed light on variations in treatment response as well as the burden of disease among RA adults [30, 31]. Our findings suggest that collecting patient-reported HRQoL can be critical in assessing disease burden that may not be captured by clinical assessment alone [30].

Depression in working-age adults with RA was associated with substantial direct and indirect economic burden. For example, the presence of depression more than doubled the annual per person total healthcare costs, a number of missed work days, and lost wages due to missed work days, even after controlling for predisposing factors, enabling characteristics, need factors, and personal health care practices. Although published evidence on the incremental economic impact of depression in RA is limited [9], our findings which are consistent with studies assessing the burden of depression on other chronic illnesses such as diabetes, cardiovascular disease, and asthma have also reported the synergistic effect of depression in increasing the economic burden among individuals with chronic conditions [12, 32].

Our findings on the economic burden of depression in RA patients have important implications for the payers as it highlights an opportunity for reducing expenditures in RA patients by increasing efforts towards screening and effectively treating depression in RA patients. Potential strategies could be improving the integration of mental health services with rheumatology practice and facilitating mental health training for rheumatologists. Future studies need to explore whether treatment for depression provides an opportunity to reduce direct healthcare expenditures associated with depression in RA patients.

The study findings have important implications for the employers because depression costs US employers more than $31 billion annually due to missed work and decreased work performance [33]. One study done in the US reported that depression leads to the highest reduction in work performance and the highest employer burden relative to any other chronic conditions [34]. Strategies that employers may adopt to improve mental health in employees include organizing workplace health promotion programs and stress management projects, which have shown the benefits of prevention and management of depression in workplace [35, 36].

To the best of our knowledge, this is the first population-based study that comprehensively examined the excess clinical, humanistic, and economic burden of depression in working-age adults with RA. Other strengths of this study include the use of nationally representative survey, adjustment of a comprehensive list of confounders such as predisposing factors, enabling factors, need factors, and personal health care practices and the use of robust statistical techniques such as GLM, and recycled prediction in estimating the incremental costs and missed workdays.

However, the findings of this study should be interpreted considering its potential limitations. First, we did not control for the severity and duration of RA and depression as MEPS does not contain this information. These factors can be important confounders of both healthcare costs and work absence. Second, we have measured productivity loss as missed work days and did not consider other kinds of productivity loss such as reduced productivity while at work (presenteeism) and loss of employment.

Our findings would provide valuable insights to payers and other decision-makers to better understand the economic impact of comorbid depression on working RA patients from US societal perspective. It is well-documented that depression in RA patients is often underrecognized and undertreated in routine clinical practice [37, 38]. Therefore, our study underscores the need for incorporating depression screening and management in the routine clinical management of RA in order to offset the substantial incremental costs associated with depression. Published evidence has well documented that depression is a treatable condition. However, it is still not clear whether depression treatment is equally effective in RA patients as compared to those with depression without RA [39]. Future studies need to assess the potential cost reductions that can be achieved through early detection and more aggressive treatment of depression in RA patients.

## Figures and Tables

**Table 1:** Description of study sample. Working-age (18 to 64 years) adults with Rheumatoid Arthritis Medical Expenditure Panel Survey (2009, 2011, 2013, and 2015).

	*N*	Weighted *N*	Weighted%
All	2,662	5,719,998	100.0
Gender			
Female	1,826	3,661,958	64.0
Male	836	2,058,040	36.0
Race/ethnicity			
White	1,061	3,596,249	62.9
African American	778	989,822	17.3
Latino	639	780,416	13.6
Others	184	353,511	6.2
Age in years			
18–39 years	444	956,652	16.7
40–49 years	609	1,271,670	22.2
50–59 years	1,080	2,326,292	40.7
60–64 years	529	1,165,384	20.4
Marital status			
Married	1,287	3,098,420	54.2
Widow/separated/divorced	845	1,630,180	28.5
Never married	530	991,398	17.3
Education			
Less than high school	665	1,007,827	17.6
High school	939	2,043,963	35.7
More than high school	1,036	2,623,400	45.9
Poverty status			
Poor	740	1,216,767	21.3
Not poor	675	1,204,168	21.1
Middle income	708	1,670,010	29.2
High income	539	1,629,054	28.5
Insurance status			
Private	1,302	3,411,533	59.6
Public	932	1,580,322	27.6
Uninsured	428	728,144	12.7
Employment			
Employed	1,309	3,071,100	53.7
Not employed	1,352	2,647,283	46.3
Region			
Northeast	383	984,779	17.2
Midwest	523	1,264,228	22.1
South	1,190	2,454,395	42.9
West	566	1,016,597	17.8
Perceived physical health			
Excellent/very good	607	1,488,614	26.0
Good	871	2,001,187	35.0
Fair/poor	1,184	2,230,197	39.0
Perceived mental health			
Excellent/very good	607	1,488,614	26.0
Good	871	2,001,187	35.0
Fair/poor	1184	2,230,197	39.0
Multimorbidity			
RA only	716	1,605,886	28.1
Multimorbidity	1,946	4,114,112	71.9
Anxiety			
Yes	494	1,134,341	19.8
No	2,168	4,585,657	80.2
Obesity			
Obese	1,218	2,521,340	44.1
Not obese	1,392	3,084,702	53.9
Smoking status			
Current smoker	698	1,567,457	27.4
Others	1,755	3,729,925	65.2
Missing	209	422,616	7.4
Exercise			
Yes	1,077	2,364,802	41.3
No	1,568	3,321,567	58.1

*Note*. Based on 2,662 adults with Rheumatoid Arthritis, aged between 18 and 64 years, who were alive during the calendar year. Missing data for the variables, education, obesity, smoking, and exercise, are not presented. Weighted *N* and percentages were derived by dividing the person weights by the number of years pooled.

**Table 2:** Description of study sample by depression among working-age (18–64 years) adults with Rheumatoid Arthritis Medical Expenditure Panel Survey (2009, 2011, 2013, and 2015).

	RA with depression			RA without depression			Sig
	*N*	Wt. *N*	Wt. Row%	*N*	Wt. *N*	Wt. Row%	
All	647	369,136		2,015	1,060,864		

Gender							*∗∗∗*
Female	502	270,895	29.6	1,324	644,595	70.4	
Male	145	98,241	19.1	691	416,269	80.9	
Race/ethnicity							*∗∗∗*
White	328	264,578	29.4	733	634,485	70.6	
African American	157	45,643	18.4	621	201,813	81.6	
Latino	127	40,938	21	512	154,166	79	
Others	35	17,977	20.3	149	70,400	79.7	
Age in years							
18–39 years	98	58,925	24.6	346	180,238	75.4	
40–49 years	149	82,063	25.8	460	235,855	74.2	
50–59 years	281	156,442	26.9	799	425,131	73.1	
60–64 years	119	71,706	24.6	410	219,640	75.4	
Marital status							*∗∗∗*
Married	248	160,488	20.7	1,039	614,118	79.3	
Widow/separated/divorced	259	139,902	34.3	586	267,643	65.7	
Never married	140	68,746	27.7	390	179,104	72.3	
Education							
Less than high school	170	65,953	26.2	495	186,004	73.8	
High school	232	138,615	27.1	707	372,376	72.9	
Missing	4	2,236	20	18	8,966	80	
Poverty status							*∗∗∗*
Poor	236	103,612	34.1	504	200,580	65.9	
Not poor	185	88,172	29.3	490	212,870	70.7	
Middle income	141	101,112	24.2	567	316,391	75.8	
High income	85	76,241	18.7	454	331,023	81.3	
Insurance status							*∗∗∗*
Private	235	183,245	21.5	1,067	669,638	78.5	
Public	345	153,607	38.9	587	241,474	61.1	
Uninsured	67	32,284	17.7	361	149,752	82.3	
Employment							*∗∗∗*
Employed	195	132,391	17.2	1,114	635,384	82.8	
Not employed	452	236,745	35.8	900	425,076	64.2	
Region							
Northeast	89	50,671	20.6	294	195,524	79.4	
Midwest	157	93,289	29.5	366	222,768	70.5	
South	271	161,001	26.2	919	452,598	73.8	
West	130	64,175	25.3	436	189,975	74.7	
Perceived physical health status							*∗∗∗*
Excellent/very good	72	46,019	12.4	535	326,135	87.6	
Good	165	110,972	22.2	706	389,325	77.8	
Fair/poor	410	212,145	38	774	345,405	62	
Perceived mental health status							*∗∗∗*
Excellent/very good	113	74,855	11.7	995	565,345	88.3	
Good	207	122,356	25.1	730	365,098	74.9	
Fair/poor	327	171,925	56.9	290	130,422	43.1	
Multimorbidity							*∗∗∗*
RA only	110	69,703	17.4	606	331,768	82.6	
Multimorbidity	537	299,433	29.1	1,409	729,096	70.9	
Anxiety							*∗∗∗*
Yes	259	150,809	53.2	235	132,776	46.8	
No	388	218,327	19	1,780	928,088	81	
Obesity							*∗∗*
Obese	353	190,757	30.3	865	439,579	69.7	
Not obese	287	172,382	22.4	1,105	598,794	77.6	
Smoking status							*∗∗∗*
Current smoker	250	142,876	36.5	448	248,988	63.5	
Others	355	204,588	21.9	1,400	727,893	78.1	
Exercise							*∗∗∗*
Yes	190	106,306	18	887	484,895	82	
No exercise	454	259,932	31.3	1,114	570,460	68.7	

*Note*. Based on 2,662 adults with Rheumatoid Arthritis aged between 18 to 64 years, who were alive during the calendar year. Missing data for the variables, education, obesity, smoking, and exercise, are not presented. Asterisks represent significant group differences by the presence of depression based on chi-square tests. Weighted *N* and percentages were derived by dividing the person weights by the number of years pooled; Wt.: weighted; ^*∗∗∗*^*p* < .001; .001 ≤ ^*∗∗*^*p* < .01.

**Table 3:** Clinical outcome associated with depression among working-age adults with Rheumatoid Arthritis Medical Expenditure Panel Survey (2009, 2011, 2013, and 2015).

	RA + depression		RA and no depression		Sig
	*N*	Wt. col%	*N*	Wt. col%	
All	605		1,875		

Pain interference with daily activity					*∗∗∗*
Mild/none	155	29.8	918	51.9	
Moderate	92	15.4	317	17.3	
Severe (extreme/quite a lot)	358	54.8	640	30.8	
Limitations					
Any functional limitations	528	79.0	1,026	51.1	*∗∗∗*

Adjusted odds ratio and 95% CI for depression from multinomial logistic regression on pain interference with normal activity					
	AOR	95% CI		Sig	

Pain interference with daily activity					
Mild/none (reference group)					
Moderate	1.37	[0.91,2.06]			
Severe	2.22	[1.55,3.18]		*∗∗∗*	

Adjusted odds ratio and 95% CI for depression from logistic regression on limitations					
	AOR	95% CI		Sig	

Limitations					
Any functional limitations	2.24	[1.62,3.10]		*∗∗∗*	

*Note*. Based on 2,662 adults with Rheumatoid Arthritis aged between 18 and 64 years, who were alive during the calendar year. Adjusted multinomial logistic regression controlled sex, race/ethnicity, age, region, marital status, education, family poverty status, health insurance, physical health status, mental health status, anxiety, multimorbidity, obesity, physical activity, and smoking. Asterisks represent significant group differences by the presence of depression; ^*∗∗∗*^*p* < .001; ADL: activities of daily living; Col: column; IADL: instrumental activities of daily living; Wt.: weighted.

**Table 4:** Humanistic outcomes (health-related quality of measures) by presence of depression among working-age adults with Rheumatoid Arthritis Medical Expenditure Panel Survey (2009, 2011, 2013, and 2015).

	RA + depression		RA and no depression		Sig
	Wt. mean	SE	Wt. mean	SE	
All	*N* = 647		*N* = 2,015		

Physical component summary score	35.07	0.89	40.18	0.47	*∗∗∗*
Mental component summary score	37.20	0.77	48.74	0.37	*∗∗∗*

Fully adjusted model: parameter estimates and standard errors for depression ordinary least squares regression mental component summary score					
	Beta	Standard error			Sig

Depression	−8.72	0.81			*∗∗∗*
No depression (reference group)					

Fully adjusted model: parameter estimates and standard errors for depression ordinary least squares regression physical component summary score					
	Beta	Standard error			Sig

Depression	−1.29	0.81			
No depression (reference group)					

*Note*. Based on 2,662 adults with Rheumatoid Arthritis aged between 18 and 64 years, who were alive during the calendar year. Asterisks represent significant group differences by the presence of depression. The ordinary least squares regressions controlled for the following variables: sex, race/ethnicity, age, region, marital status, education, family poverty status, health insurance, anxiety, multimorbidity, obesity, physical activity, and smoking; SE: standard error; Wt.: weighted; ^*∗∗∗*^*p* < .001.

**Table 5:** Economic outcomes by presence of depression among working-age adults with Rheumatoid Arthritis Medical Expenditure Panel Survey (2009, 2011, 2013, and 2015).

	RA + depression		RA and no depression		Sig
	Wt. mean	SE	Wt. mean	SE	
All	*N* = 647		*N* = 2,015		

Total healthcare expenditures (2015 $)	17,941	1489	10,064	574	*∗∗∗*
Total out-of-pocket spending by patients/families (2015 $)	$1,443	135	$1,052	73	*∗∗∗*

Adjusted total direct healthcare expenditures of depression from generalized linear models with gamma distribution and log link					
	Wt. mean	95% CI	Wt. mean	95% CI	Sig

					*∗∗∗*
Total healthcare expenditures (2015 $)	14,752	(14,411–15,125)	10,541	(10,206–10,806)	
Total out-of-pocket spending by patients/families (2015 $)	1,232	(1,202–1265)	979	(955–1,005)	*∗∗∗*

Incremental total direct healthcare expenditures of depression from counterfactual recycled prediction					
	Wt. mean	95% CI			

Total healthcare expenditures (2015 $)	4,212	(4,114, 4318)			*∗∗∗*
Total out-of-pocket spending by patients/their families (2015 $)	253	(247–260)			*∗∗∗*

High out-of-pocket spending burden (>10% income spent on healthcare)					
	RA + depression		RA and no depression		
	*N*	Wt. col%	*N*	Wt. col%	

High out-of-pocket spending burden	192	30.7	424	21.3	*∗∗∗*

Fully adjusted model: adjusted odds ratio (AOR) and 95% confidence intervals (CI) of Depression from logistic regression on high out-of-pocket burden					
	AOR	95% CI	Sig		

Depression	1.34	[1.01,1.79]			*∗∗*
No depression (reference)					

Unemployment among working-age adults					
	RA + depression		RA and no depression		
	*N*	Wt. col%	*N*	Wt. col%	

Unemployed	452	64.1	900	40.1	*∗∗∗*

Fully adjusted model: adjusted odds ratio (AOR) and 95% confidence intervals (CI) of depression from logistic regression on unemployment					
	AOR	95% CI	Sig		

Depression	1.55	[1.14, 2.10]	*∗∗∗*		
No depression (reference)					

Fully adjusted models: total productivity losses by depression from negative binomial regression on missed work Days					
	Wt. mean	95% CI	Wt. mean	95% CI	Sig

					*∗∗∗*
Number of missed work days	9.0	(8.7–9.4)	6.0	(5.7–6.2)	
Lost wages	853	(833–873)	571	(558–584)	*∗∗∗*

Incremental total productivity losses associated with depression from counterfactual recycled prediction					
	Wt. mean	95% CI			

Number of missed work days	3.1	(2.9–3.2)			*∗*
Lost wages	282	(276–289)			*∗*

*Note*. Based on 2,662 adults with Rheumatoid Arthritis aged between 18 and 64 years, who were alive during the calendar year. Asterisks represent significant group differences by the presence of depression. The adjusted models squares regressions controlled for the following variables: sex, race/ethnicity, age, region, marital status, education, family poverty status, health insurance, physical health, mental health status, anxiety, multimorbidity, obesity, physical activity, and smoking. Missed work days and lost wages were estimated only for those who were employed; ^*∗∗∗*^*p* < .001; .001 ≤ ^*∗∗*^*p* < .01; .01 ≤ ^*∗*^*p* < .05.
